# Impact of aging experience suit on neuropsychological performance

**DOI:** 10.3389/fpsyg.2025.1568296

**Published:** 2025-08-04

**Authors:** Pablo Medrano-Martínez, Isabel Carretero, Cristina Noriega, Javier López, Gema Pérez-Rojo

**Affiliations:** Grupo de Investigación BUENAVEJEZ, Departamento de Psicología y Pedagogía, Facultad de Medicina, Universidad San Pablo-CEU, CEU Universities, Campus de Montepríncipe, Alcorcón, Madrid, Spain

**Keywords:** older adults, aging, simulation technology, neurocognitive assessment, cognitive performance

## Abstract

**Introduction:**

Although the effect of Aging experience suit (AES) interventions in the increase of empathy and positive attitudes toward older adults has been well studied, the evidence about the influence of the AES in neuropsychological performance remains limited. The aims of this study were to assess the effect of wearing an AES during neuropsychological evaluation and describe how physical and visuoperceptual restrictions could affect the interpretation of the results.

**Methods:**

We studied 48 subjects (72.9% females; mean age, 19.50 ± 0.61 years). All participants completed two testing sessions: one wearing the AES and another without it. They underwent neuropsychological tests for attention and executive function, Trail Making Test (TMT) and Cancellation Task, and completed a dual task. The dual-task conditions included walking and backward counting simultaneously. Heart rate was recorded to measure fatigue.

**Results:**

Participants demonstrated poorer performance on both, TMT-A and TMT-B while wearing the AES, however no significant differences were observed in the derived TMT indices. In the cancellation task, AES condition participants processed fewer stimuli and achieved less correct responses. In the dual-task assessment, performance while wearing the suit decreased significantly. Participants appeared to prioritize the cognitive component (backward counting) over the motor task (walking), as evidenced by the reduced walking distance, suggesting a shift in attentional focus toward the secondary task.

**Conclusion:**

The results of the neuropsychological tests indicated generalized poorer performance in the suit condition. AES leads to an impaired performance on standardized neuropsychological tests.

## 1 Introduction

Demographic trends indicate a strong increase in the older population group that will continue in the coming years (Levy et al., 2022). The increase in life expectancy raises concerns about the perception of aging. Numerous studies have reflected the high prevalence of predominantly negative attitudes and beliefs toward older adults (World Health Organization (WHO), 2021).

The Aging simulation equipment allows students to gain first-hand insight into the physical and sensory challenges commonly experienced by older adults (Gaba, 2004; Nelson et al., 2023). Numerous hospitals and universities use this type of equipment (Tremayne et al., 2011). The impact of the Aging simulation equipment is achieved by external elements to limit mobility and sensory capabilities. Some suits have been used to measure the physical limitations of aging on a specific part of the body such as the feet (Eymard et al., 2010) or the hands (O’Brien et al., 2015). The GERonTological Simulator is a relatively new suit (Moll, 2019) that allows users to simultaneously experience different limitations commonly associated with aging. The aging limitations that can be experienced by students are visual (eye lens opacity, visual field restriction), auditory (high frequency deafness), restricted head mobility, joint stiffness, loss of strength, reduced grasping ability and reduced coordination (Moll, 2019). To our knowledge, there are no studies comparing the effectiveness of different suits as ageing simulation tools.

Some studies have shown how the Aging Experience Suit (AES) and similar equipment allows students to experience the challenges faced by older adults when performing basic everyday tasks in a safe and controlled environment (Nelson et al., 2023), increasing professionals’ empathy and understanding of aging related issues, which contributes to more inclusive and compassionate perspectives (Bouwmeester Stjernetun et al., 2023; Fernández-Gutiérrez et al., 2022). A recent systematic review further highlights the significance of interventions involving AES and similar technologies (Gerhardy et al., 2022).

Although the effect of AES in empathy has been well studied (Bouwmeester Stjernetun et al., 2023), the evidence about its influence in neuropsychological performance is scarce. Enhancing knowledge about the effects of aging simulations on neuropsychological performance is crucial for fostering empathy and reducing age-related biases. Such simulations not only illuminate the physical limitations experienced by older adults but also bring attention to the often-overlooked cognitive challenges, such as memory decline, reduced processing speed, and difficulties with attention and executive functioning. By gaining a more comprehensive understanding of both cognitive and physical aspects of aging, healthcare professionals can develop more empathetic, informed, and person-centered approaches to care.

To date, only two studies have focused on how AES simulate older adults’ physical and visuoperceptual impairments using neuropsychological assessments (Vieweg et al., 2023; Vieweg and Schaefer, 2020). Both studies have used the GERT aging suit. To our knowledge, no other AES have been used in the assessment of cognitive functioning. Vieweg and Schaefer (2020) found a significant difference in the Digit Symbol Substitution test (DSST) performance between wearing and not wearing an AES. However, a similar study by the same research group did not replicate these findings, observing no significant differences between conditions (Vieweg et al., 2023). Additionally, the authors evaluated information processing speed using a reaction time task but did not find significant differences. However, fine motor skills were notably impaired, with performance levels resembling those typically observed in people in their early 80s (Vieweg and Schaefer, 2020). Using the suit as a simulation tool restricts movement execution (Lavallière et al., 2017) and impairs perceptual processing through the use of gloves and glasses (Vieweg et al., 2023). While it effectively interferes with information processing by limiting both perception (e.g., visual acuity) and mobility, cognitive processing itself seems to remain largely intact.

These contradictory findings empathize the relevance of exploring further the impact of AES on neuropsychological performance. Moreover, several commonly used cognitive assessments remain controversial due to ongoing debates about the specific cognitive functions they measure. The lack of consensus regarding the underlying processes involved in task performance, has led to the development of various indices aimed at isolating distinct cognitive processes. In this context, AES may serve as a valuable tool for disentangling the effects of physical and visuoperceptual impairments on cognitive task performance, offering new insights into how such limitations influence neuropsychological outcomes. Previous studies have also examined the impact of different secondary tasks on dual-task gait performance in older adults (Al-Yahya et al., 2011). Findings suggest that older adults respond differently to increased dual-task demands depending on the nature of the secondary cognitive task. For instance, during mental tracking tasks performed while walking, older adults tend to prioritize gait over cognitive task (Hui-Ting et al., 2021). However, studies involving young adults using the AES, have not reported similar patterns of interference, with the cognitive task not significantly affecting motor performance (Gerhardy et al., 2024). This raises the question of whether young adults under simulated aging conditions exhibit behavioral patterns comparable to those of older adults when task difficulty increases.

While some studies have focused on studying the impact of AES on motor tasks (Al-Yahya et al., 2011), to date only two studies have assessed the impact of AES on performance in neuropsychological test, and both involved small sample sizes (Vieweg et al., 2023; Vieweg and Schaefer, 2020). Elucidating how physical and visuoperceptual impairments influence cognitive test performance in university students wearing AES, would help to distinguish whether performance deficits arise from genuine cognitive impairment or are instead due to external limitations imposed by the suit. The study examines the influence of wearing the AES on performance in neuropsychological tests, allowing us to determine the extent to which physical limitations affect cognitive task performance. To test this, the aims were as follows: (1) to assess the effect of wearing AES in the Trail Making Test (TMT) and cancellation task performance and (2) to assess how high difficulty cognitive tasks influence the performance on a dual-task gait condition. We hypothesized that emerging adulthood wearing the AES will show reduced performance in TMT and cancellation task, despite having no cognitive impairment. This decline is expected to be due to the physical and perceptual limitations imposed by the AES rather than cognitive impairment. Additionally, when wearing the AES, emerging adulthood may exhibit behavioral patterns similar to those of older adults; in particular, a shift in attentional resources that favours gait maintenance over cognitive performance in dual-task conditions.

## 2 Methods

### 2.1 Participants

The study population comprised 48 subjects (72.9% females; mean age, 19.50 ± 0.61 years; age range 18–21) from Madrid and the metropolitan area (Spain). The gender distribution is consistent with that observed in health sciences programs at other universities across the country (UNIVbase, 2025). Their body mass index (BMI) was 22.86 ± 3.04. Participants were recruited from the early years of various health science degree programs, at the same university, ensuring a similar educational background.

The inclusion criteria were the following: (a) enrollment in the first 2 years of a health science degree program and (b) provision of signed informed consent.

The exclusion criteria were the following: (a) reported history of cardiovascular disease or musculoskeletal dysfunctions; (b) being under 18-year-old. Three subjects were excluded from the study for not meeting these criteria.

### 2.2 Age simulation suit

The age simulation suit GERT (Moll, 2019) was used to simulate the consequences of physical and sensory aging. To replicate physical decline the suit increased 20 kg the weight of participants, impairing strength and coordination. The additional weight was distributed as follows: 1.5 kg on each wrist, 2.3 kg on each ankle and 10 kg in an upper-body vest. Overshoes were used to reduce balance and insecure the gait. Also, a neck ruff restricted head mobility. Sensory impairments were simulated using colored glasses, which altered visual processing, and earmuffs, which reduced auditory input.

### 2.3 Variables and instruments

#### 2.3.1 Neuropsychological assessment

##### 2.3.1.1 Trail making test

The TMT consists of two parts (Reitan, 1992). In Part A (TMT-A), participants are instructed to connect 25 numbered circles in ascending order. In Part B (TMT-B), they must alternately connect 25 circles containing numbers and letters in sequential order (e.g., 1-A-2-B, etc.) (Lezak et al., 2012). In accordance with Reitan’s guidelines, errors are corrected immediately as they occur, allowing the participant to complete the test without leaving any mistakes uncorrected. TMT-A primarily assesses processing speed and visuoperceptual abilities, while TMT-B evaluates working memory and cognitive flexibility, particularly task-switching (Sánchez-Cubillo et al., 2009). In both parts, the time required to complete the task correctly was recorded. Additionally, the B–A index (i.e., the difference in completion time between TMT-B and TMT-A) (Lezak et al., 2012), and the B/A ratio (i.e., the ratio of completion times between TMT-B and TMT-A) (Lamberty et al., 1994), were calculated to control for the influence of basic visuoperceptual and motor speed components on executive performance.

##### 2.3.1.2 Cancellation test

The cancellation test (4th edition of the Wechsler Adult Intelligence Scale) requires participants to cross out, within 45 s, all geometric figures that match both the shape and color of the target stimuli. The test includes a total of 180 stimuli of which 36 are target. The test assesses selective attention, processing speed and visuoperceptual abilities. It was administered according to the standard protocol (Wechsler, 2012). Correct stimuli, omissions, false positives, and total stimuli processed were registered for analysis.

##### 2.3.1.3 Dual task

A dual task paradigm was conducted to assess the impact of cognitive load on gait performance. The procedure followed the counting backward protocol described by Hui-Ting et al. (2021) and was classified as mental tracking according to Al-Yahya et al. (2011). Initially participants are instructed to walk in a straight line along a predefined 6-metre path in an unobstructed room. Upon reaching the end, they performed a 180° turn and continued walking back and forth along the same path for 30 s. The distance covered during this period is measured and used as the baseline. Following this, participants are given a starting number and asked to count backward by sevens for 1 min and 30 s while continuing to walk. This extended duration is intended to progressively increase the task’s difficulty (McIsaac et al., 2015). Every 30 s, the participant pauses briefly so the distance walked during the interval can be recorded. Throughout the task, several walking-related variables are measured, including the total distance covered during the countdown and any pauses or stops made. Additionally, the accuracy of the counting task is assessed by recording both correct responses and errors. In addition, the dual task cost (DTC) was calculated according to Kelly et al. (2010).

##### 2.3.1.4 Heart frequency

The heart frequency was also recorded, as a measure of fatigue, using a portable device. The Beurer PO30 Pulse Oximeter was used for this purpose. Heart frequency was monitored for 1 min, and the value was recorded once it remained stable for at least 5 s. Two measurements were taken: the resting measurement was taken with the participant wearing the AES before performing any tasks. The second measurement was taken at the end of the second dual-task trial, also while the participant was wearing the AES. In both cases, the participant remained standing in a stationary position without movement.

### 2.4 Procedure

Neuropsychological testing was performed between 9:00 a.m. and 1:00 p.m. The approximate duration of the assessment was 40 min for the two sessions; no participant requested a break between sessions. All neuropsychological tests included three practice sessions which were administered during both sessions to ensure familiarity with the task. The dual task was divided into three 30-s trials to examine whether the effects of the suit were consistent over time and whether younger participants were able to adapt and compensate for the limitations imposed by the suit on repeated trials. Following a repeated-measures design, all participants completed two testing sessions: one wearing the AES and another not wearing it. Heart frequency was registered only in the age simulation suit condition using the Beurer PO30 Pulse Oximeter. Measurements were taken during a resting state and again at the conclusion of the second dual-task trial to assess physical exertion.

To control practice effects in test performance, the order of testing sessions was counterbalanced across participants. Additionally, an alternate version of TMT was used to minimize learning effects (Wagner et al., 2011). For the Cancellation test (Wechsler, 2012) which includes two similar tasks with varying target stimuli, the version administered in each condition was randomized. In the dual task the starting number for counting backward differed (900 vs. 500) between sessions and was randomly assigned.

To further reduce the potential effects of fatigue on performance, the presentation order of the neuropsychological test was randomized within each condition.

### 2.5 Data analysis

Continuous variables are expressed as mean ± SD, skewness and kurtosis were also included. Categorical variables are expressed as frequency and percentage.

The analyses of the neuropsychological assessment with and without the AES were tested using dependent *t*-tests. Cohen’s *d* was reported as a measure of the effect size, with values of 0.41, 1.15 and 2.70 suggesting, respectively, a minimum, moderate and strong effect size (Cohen, 1992; Ferguson, 2009).

The dual task performance with and without the AES was tested using analyses of variance (ANOVA repeated measures), with suit use as between-subjects factor, and performance in the three different trials (3) as within-subjects factors. For all ANOVAs, *F* values and η_p_^2^ for effect sizes were reported. If sphericity assumption were not met, Greenhouse–Geisser test was reported. A *post hoc* analysis was conducted using Bonferroni correction.

Statistical Analysis were performed using SPSS Statistics for Windows version 27.0.

## 3 Results

Table 1 summarizes the scores achieved in the neuropsychological tests.

**Table 1:** Results of the neuropsychological assessment.

	No AES (*n* = 48)			AES (*n* = 48)			*t*-value	*p*-value	*d*
	Mean ± SD	Skewness	Kurtosis	Mean ± SD	Skewness	Kurtosis			
Trail making test									
TMT-A (sec)	23.31 ± 8.9	2.29	6.82	28.69 ± 9.89	0.74	0.48	−3.52	0.00^*^	−0.57
TMT-B (sec)	51.39 ± 18.58	6.82	1.60	64.79 ± 27.52	2.76	11.54	−3.22	0.00^*^	−0.57
TMT B−A	28.07 ± 16.87	1.73	4.59	36.10 ± 26.81	2.76	11.94	−1.99	0.05	−0.35
TMT B/A	2.33 ± 0.94	2.60	9.79	2.42 ± 1.05	1.47	3.16	−0.53	0.59	−0.92
Cancellation test									
Correct stimuli	22.40 ± 4.10	0.22	0.09	20.54 ± 4.37	0.40	−0.70	4.04	0.00^*^	0.43
False positives	0.0 ± 0.0	–	–	0.4 ± 0.20	4.73	21.32	−1.43	0.15	−0.29
Omissions	1.17 ± 1.47	1.97	4.72	1.29 ± 1.32	0.67	−0.64	−0.45	0.65	−0.88
Total stimuli processed	115.40 ± 25.49	0.84	0.87	108.40 ± 26.73	0.32	0.70	2.29	0.02^*^	0.26

Correct stimuli refer to the number of accurate responses in the cancellation task. False positives indicate the number of times the participant marked a non-target stimulus, while omissions represent the number of target stimuli that were not marked within the given time. The total number of stimuli processed corresponds to the final item the participant reached before the time limit expired. AES, Age Experience Suit; TMT-A, Trail Making Test—Part A; TMT-B, Trail Making Test—Part B; TMT B−A, Trail Making Test Difference; TMT B/A, Trail Making Test ratio. ^*^Significant differences if *p* < 0.05.

### 3.1 Trail making test

Significant differences were observed in both TMT-A (*t* = −3.52; *p* = 0.00) and TMT-B (*t* = −3.22; *p* = 0.00) indicating that participants required more time to complete both TMT parts while wearing the AES compared to not wearing it. However, no significant differences were found in the derived measures designed to minimize visuoperceptual demands: the difference in completion times between TMT-B and TMT-A (TMT B−A; *t* = −1.99; *p* = 0.05) and the ratio of completion times (TMT B/A; *t* = −0.53; *p* = 0.59). These results suggest that the increased completion times in the AES condition may be primarily attributed to physical or perceptual constraints rather than cognitive flexibility alone. The effect sizes for TMT-A (*d* = −0.57) and TMT-B (*d* = −0.57) exceed the recommended threshold for assuming a clinically significant effect (see Table 1).

### 3.2 Cancellation test

In the Cancellation test, subjects wearing AES performed significantly worse than when not wearing it. They identified fewer correct target stimuli (*t* = 4.04; *p* = 0.00) and processed a lower total number of stimuli (*t* = 2.29; *p* = 0.02). The effect size is considered minimum for the number of correct responses (*d* = 0.43) and for the total number of stimuli processed (*d* = 0.28) suggesting a small yet meaningful impact of the AES on task performance. In contrast, they committed similar false positives (*t* = −1.43; *p* = 0.15), or omissions (*t* = −0.45; *p* = 0.65) in both conditions.

### 3.3 Dual task

**Figure 1:**
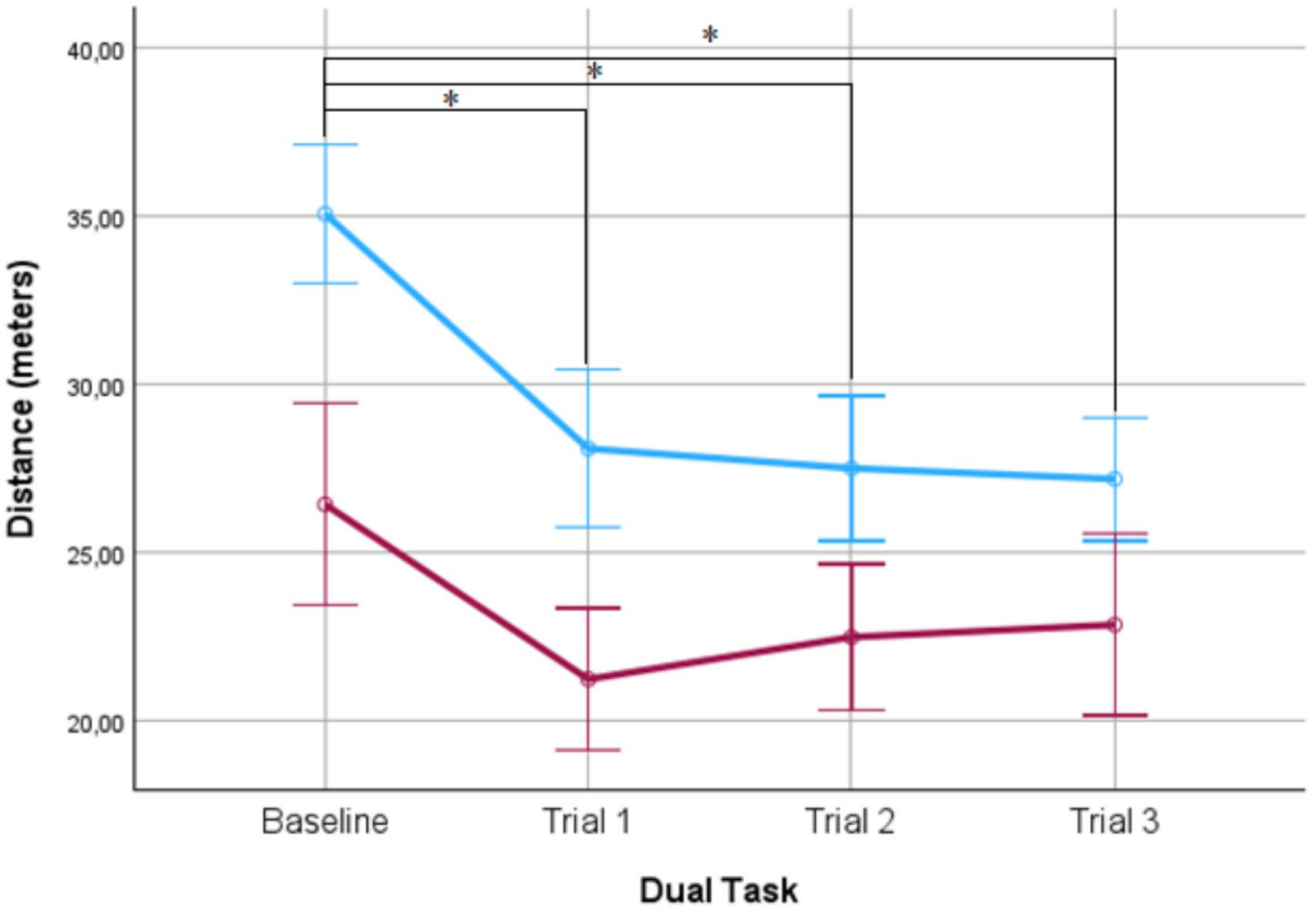
Distance covered with (red) and without (blue) the Aging Experience Suit (AES). Significant differences were also found in the distance covered in each of the three trials based on wearing AES. Error bars ± 2SD.

The scores obtained in the dual task are shown in Table 2. The ANOVA repeated measures revealed a significant effect only for the distance covered during the dual task walking condition. No differences were found between conditions in the number of stops, errors or correct responses. Simple main effects analysis revealed a significant effect of wearing AES on walking performance (*F* (1,48) = 54.39; *p* < 0.00; η_p_^2^ = 0.54) as well as a significant effect of trial progression on the distance covered across the four different trials (*F* (3,48) = 16.82; *p* < 0.00; η_p_^2^ = 0.27). The interaction between these two variables was significant (F (3,48) = 3.02; *p* < 0.03; η_p_^2^ = 0.06). *Post hoc* analyses revealed that participants wearing AES walked slower than not wearing it in all trials (*p* < 0.00) and that performance was significant better in the baseline compared with the three following trials (*p* < 0.00) (see Table 2). Significant differences were also found in the distance covered in trial one (*t* = 5.36; *p* < 0.00; *d* = 9.33), trial two (*t* = 4.30; *p* < 0.00; *d* = 8.03), trial three (*t* = 3.60; *p* < 0.00; *d* = 8.15) and baseline (*t* = 6.38; *p* < 0.00; *d* = 9.52) based on wearing AES (see Figure 1). No significant differences were found in DTC score comparison (*t* = 0.51; *p* = 0.60) between wearing AES (mean: 7.29 ± 46.55; range: 471.42) and not wearing AES (mean: 13.27 ± 66.70; range: 324.24).

**Table 2:** Results of dual task assessment.

	No AES (*n* = 48)			AES (*n* = 48)			*f*-value	*p*-value	η_p_^2^
	Mean ± SD	Skewness	Kurtosis	Mean ± SD	Skewness	Kurtosis			
Correct responses on backward task									
1st trial	5.17 ± 3.96	1.61	3.31	4.48 ± 3.35	1.17	1.33	0.84	0.43	0.01
2nd trial	5.55 ± 4.07	1.27	1.76	5.38 ± 3.37	1.19	2.68			
3rd trial	5.26 ± 3.45	1.28	1.89	5.23 ± 3.58	0.71	0.15			
Errors on backward task									
1st trial	1.79 ± 2.52	1.91	4.60	1.44 ± 2.07	1.74	2.77	0.24^a^	0.76	0.00
2nd trial	1.60 ± 2.39	1.75	2.81	1.06 ± 1.88	3.49	16.18			
3rd trial	1.33 ± 2.20	1.91	3.11	1.06 ± 1.37	1.57	2.51			
Distance covered (meters)									
Baseline	35.14 ± 6.97	−1.53	5.49	26.36 ± 10.03	1.05	3.19	3.02	0.03^*^	0.06
1st trial	28.58 ± 8.63	1.86	5.14	21.34 ± 7.04	0.48	0.44			
2nd trial	27.54 ± 7.27	0.65	0.19	22.53 ± 7.22	1.06	2.19			
3rd trial	27.17 ± 6.21	0.49	−0.37	22.88 ± 8.95	2.58	10.81			
Stops on the march									
Baseline	0.00 ± 0.00	–	–	0.00 ± 0.00	–	–	1.76^a^	0.18	0.03
1st trial	0.11 ± 0.37	3.82	15.30	0.27 ± 0.64	2.68	7.39			
2nd trial	0.26 ± 0.57	2.18	3.79	0.21 ± 0.61	3.24	10.54			
3rd trial	0.11 ± 0.37	3.77	14.91	0.25 ± 0.52	2.06	3.57			

Correct responses and errors in the backward counting task reflect performance on the cognitive component during each recorded trial. The distance covered corresponds to the number of meters walked during the dual-task condition, with both the baseline and each trial lasting 30 s. Stops on the march refer to the number of pauses made by the participant during each trial. ^*^Significant differences if *p* < 0.05.AES, Age Experience Suit.

a Greenhouse–Geisser test.

### 3.4 Heart frequency

Both conditions, resting measurement (mean: 77.67 ± 13.03 beats per minute) and the second measurement at the end of the second trial of the dual-task (mean: 80.99 ± 17.42 beats per minute) showed similar heart frequency rate (*t* = −0.84; *p* = 0.20).

## 4 Discussion

The aim of this study was to assess the effect of wearing AES in neuropsychological tests performance in emerging adulthood. The findings revealed a generalized decrease in performance when participants wore the AES.

These results were like those of a previous study that included other neuropsychological tests (Vieweg and Schaefer, 2020). Specifically, performance decrements were observed in both parts A and B of the TMT. In contrast, non-significant differences were found for the TMT index scores. This suggests that the visuomotor impairments induced by wearing the AES primarily affect tasks reliant on visuomotor speed, as seen in TMT-A and cancellation test. According to Ferguson’s (2009) criteria, the effect sizes are sufficiently large to suggest that the suit has a meaningful impact on task performance.

In the cancellation test, participants in the AES condition processed fewer stimuli and consequently achieved fewer correct responses. These results agree with those obtained by Vieweg and Schaefer (2020). In their study young participants carried out the DSST once with and once without the suit, performances were significantly lower when wearing the suit. In contrast a recent study did not find any significant differences in the DSST or figural speed tests under similar conditions (Vieweg et al., 2023). It is worth mentioning that the number of subjects in our study is larger than that of the aforementioned studies.

Taken together, the results from both the TMT and cancellation tests suggest that the AES is a reliable tool for simulating visuomotor impairments. Interestingly, the absence of differences in the TMT B−A and B/A indexes scores further supports the utility of these scores as a “pure” measure of executive function, relatively unaffected by visuomotor abilities (Sánchez-Cubillo et al., 2009). These findings, consistent with our hypothesis, emphasize the importance of using the index score when assessing executive functions independently from visuomotor abilities.

In the dual-task condition, results showed an effect of wearing AES, which contrasts with the findings reported by Gerhardy et al. (2024). This discrepancy is probably attributable to differences in cognitive task difficulty. In our study we found a cognitive-related motor interference, consistent with the classification proposed by Plummer et al. (2013). This interference was present in both conditions but was intensified by the AES, likely due to the novelty of the task. According to McIsaac et al. (2015), task novelty can elevate the difficulty of single-task components, thereby exacerbating dual-task interference. Participants prioritized the secondary cognitive task over gait, resulting in a reduction in distance covered. This behavior differs from that observed in older adults, who has been described as prioritizing gait in dual-task situations (Hui-Ting et al., 2021), but this is consistent with the results of other studies that have found an effect of the suit on distance covered (Gerhardy et al., 2025). Young participants, even wearing AES, prioritize performance in mental tracking tasks, likely because they perceive it as more challenging and stimulating. In contrast, older adults tend to prioritize the walking task over the cognitive one, which may be explained by their fear of falling and the potential health consequences associated with it. However, it would have been expected that despite the increased difficulty in the cognitive task, participants would have shown stable levels in the motor task (Schaefer, 2014).

Additionally, the overlap in the activation of indirect locomotor pathways, the frontoparietal network during the gait tasks and the frontoparietal cortical regions associated with attention and executive functions may explain the performance decrements in the motor task under both conditions (Bayot et al., 2018). Slowing gait in the AES condition may allow participants to allocate more attentional resources to the secondary cognitive task (Patel et al., 2014).

Our sample consisted of emerging adulthood, predominantly female, with a high level of education. These homogenic characteristics may moderate the impact of the AES, limiting the generalizability of our findings. Regarding the potential bias introduced by the homogenic nature of the sample, gender does not appear to significantly influence performance on the TMT (Strauss et al., 2006) or on cancellation tasks (Güven et al., 2017). Therefore, there is no compelling evidence to suggest that female participants would perform differently from males in our study. While the age range of our sample may limit the generalizability of the findings, using a sample of emerging adults—whose motor performance is typically intact—ensures the relevance of our results. Although participants were recruited from similar academic backgrounds, we do not believe this introduces bias, as performance on the tasks used in this study is not related to academic training. Furthermore, it should be noted that the AES may have a broader impact than what is reflected in the present study, particularly in domains such as visual acuity (Vieweg et al., 2023) and auditory perception, which were not specifically assessed. These aspects should be considered in future research. Additionally, there is currently no evidence to confirm that results would replicate using others aging simulation equipments, highlighting the need for more studies. In contrast, the findings related to TMT indices would have broader generality beyond emerging adulthood, if participants do not have any cognitive impairment. Although we randomized both the experimental condition, the order of the test presentation, and the use of parallel tests to minimize practice effects, we cannot entirely rule out the possibility of such effects influencing the results. We have no reason to believe that the results depend on other characteristics of the participants, materials, or context. A lack of standardized normative on some of the scales in Spanish population limits the interpretability of our results and prevents direct comparisons with the performance of older adults. Additionally, from a methodological perspective, the use of the aging suit in combination with neuropsychological tasks could serve as a useful model for studying the interaction between sensorimotor and cognitive factors. While this study highlights the importance of controlling for sensorimotor variables when interpreting cognitive test results, it is not possible to draw clinical conclusions due to the limited sample size and the lack of comparable studies. In the educational domain, although it was not the primary objective of this research, the findings support the use of the suit as a tool for raising awareness. Participants reported increased difficulty in performing tasks, which supports its effectiveness in enhancing sensitivity to the challenges associated with aging.

In conclusion, wearing an AES led to impaired performance on standardized neuropsychological tests. These findings emphasize the importance of assessing motor and visual performance prior to cognitive testing, as visuomotor deficits could potentially confound interpretations of neuropsychological results.

## Data Availability

The raw data supporting the conclusions of this article will be made available by the authors, without undue reservation.
